# Trait mindful awareness predicts inter-brain coupling but not individual brain responses during naturalistic face-to-face interactions

**DOI:** 10.3389/fpsyg.2022.915345

**Published:** 2022-09-30

**Authors:** Phoebe Chen, Ulrich Kirk, Suzanne Dikker

**Affiliations:** ^1^Psychology Department, New York University, New York City, NY, United States; ^2^Department of Psychology, University of Southern Denmark, Odense, Denmark; ^3^Department of Clinical Psychology, Free University Amsterdam, Amsterdam, Netherlands; ^4^Max Planck - NYU Center for Language Music and Emotion, New York University, New York City, NY, United States

**Keywords:** mindfulness, empathy, interpersonal relations, interbrain synchrony, social neuroscience, naturalistic interaction

## Abstract

In recent years, the possible benefits of mindfulness meditation have sparked much public and academic interest. Mindfulness emphasizes cultivating awareness of our immediate experience and has been associated with compassion, empathy, and various other prosocial traits. However, neurobiological evidence pertaining to the prosocial benefits of mindfulness in social settings is sparse. In this study, we investigate neural correlates of trait mindful awareness during naturalistic dyadic interactions, using both intra-brain and inter-brain measures. We used the Muse headset, a portable electroencephalogram (EEG) device often used to support mindfulness meditation, to record brain activity from dyads as they engaged in naturalistic face-to-face interactions in a museum setting. While we did not replicate prior laboratory-based findings linking trait mindfulness to individual brain responses (*N* = 379 individuals), self-reported mindful awareness did predict dyadic inter-brain synchrony, in theta (~5–8 Hz) and beta frequencies (~26-27 Hz; *N* = 62 dyads). These findings underscore the importance of conducting social neuroscience research in ecological settings to enrich our understanding of how (multi-brain) neural correlates of social traits such as mindful awareness manifest during social interaction, while raising critical practical considerations regarding the viability of commercially available EEG systems.

## Introduction

Recent years have seen an increase in popular interest in the benefits of mindfulness. As a personality trait, mindfulness refers to attending to the present moment experience without judging occurring feelings or thoughts (Bishop et al., 2006), and has been associated with prosocial behaviors and traits (Donald et al., 2019): Multiple psychometric studies have shown that trait mindfulness is correlated with agreeableness (Thompson and Waltz, 2007), empathy (Dekeyser et al., 2008), and conscientiousness (Thompson and Waltz, 2007; Giluk, 2009); and mindfulness-based interventions and training programs are found to effectively enhance compassion and empathy (Kreplin et al., 2018; Campos et al., 2019).

Some prevailing frameworks for understanding mindfulness theorize that the practice improves attentional control, self-awareness, metacognition, and emotional control (Lutz et al., 2007; Vago and Silbersweig, 2012). Indeed, mindfulness-related changes in brain and behavior have been observed various socially relevant behavioral paradigms, such as the Affective Stroop Task (Allen et al., 2012), pain perception tasks (Grant et al., 2011; Lutz et al., 2013; Mascaro et al., 2013), emotional provocation (Taylor et al., 2011), and prosocial decision-making (Kirk et al., 2016). These and related findings have led researchers to suggest that people who practice mindfulness develop self-regulation capacities (Chiesa et al., 2011), which in turn leads to the ability to increase the awareness of others in social settings (Schindler and Pfattheicher, 2021). Taken together, these findings suggest that by training their self-regulation functions, mindful individuals may be better able to observe and alter their social responses and emotional awareness, and engage in prosocial behaviors (Tang et al., 2015; Berry et al., 2020). This is indirectly supported by neuroimaging studies of mindfulness meditators: A meta-analysis found meditators, compared to novices, exhibited consistent changes in regions that have been associated with self-awareness (Craig, 2004), higher-order self-processing (Cavanna and Trimble, 2006), and metacognition (Christoff and Gabrieli, 2000; Fleming et al., 2010; McCaig et al., 2011). Electroencephalogram (EEG) studies, in turn, have identified lower frontal gamma activity in long-term mindfulness meditators during meditation (Lutz et al., 2004).

Crucially, however, despite mindfulness being linked to prosocial traits and to various affective modalities in controlled laboratory studies, few studies to date have investigated the neural correlates of trait mindfulness during naturalistic social interaction (Kaplan et al., 2018 ). Neural correlates of mindfulness-related prosociality have been observed using fMRI in controlled tasks like viewing emotional images (Taylor et al., 2011) and playing an Ultimatum Game (Kirk et al., 2016). In the case of naturalistic behaviors, mindfulness-related prosociality has been measured using self-report. For example, one survey study showed that trait mindfulness is associated with a heightened perceptual focus in conversations, but not daily behavioral patterns that exhibit prosocial orientation (Kaplan et al., 2018). Critically, while mindfulness meditation has been theorized to support social brain function, to our knowledge this has yet to be validated in naturalistic social settings: We are unaware of any studies that have linked neural activity during naturalistic interaction to either trait mindfulness or mindfulness-based training. To fill this gap, we recorded brain activity from dyads during face-to-face communication, and asked whether mindfulness-related traits (Brown and Ryan, 2003) predict neural responses during social interaction similar to those in laboratory-based tasks.

To answer this question, we adopted a “crowdsourcing” approach to collect naturalistic inter-brain synchrony data in a participatory art installation Mutual Wave Machine, an interactive multi-brain neurofeedback installation that translates the real-time correlation of pairs’ EEG activity into light patterns (described in detail in Dikker et al., 2021; also see wp.nyu.edu/mutualwavemachine). Pairs of museumgoers were invited to interact naturally with each other while receiving audio-visual feedback of their inter-brain synchrony. We recorded their EEG activities for the neurofeedback and for offline analyses. In the offline analyses, our group has previously found that inter-brain coupling is linked to social closeness, personal distress, and shared social attention (Dikker et al., 2017, 2021), demonstrating scientific validity of the paradigm. Here, we use the same paradigm (Chen et al., 2021) to investigate trait mindful awareness, with the following differences: we used a four-channel instead of a 14-channel portable EEG headset, adopted a different synchrony metric (Circular Correlation Coefficient, see Data Analysis), and asked different research questions.

To record participants’ brain activity we used Muse, a 4-channel EEG headband commercialized as a neurofeedback tool for mindfulness-based stress reduction training (MBSR; Hashemi et al., 2016). As a neurofeedback tool, Muse and its accompanying app have been reported to be effective in reducing stress in breast-cancer patients (Millstine et al., 2019), and improving well-being and attention (Bhayee et al., 2016). The validity of Muse-collected data was demonstrated in a couple of studies: an ERP study showed the pooled average of TP9 and TP10 electrodes successfully captured the N200, P300 responses (Krigolson et al., 2017); EEG data signatures such as power spectral density (PSD), the individual alpha frequency (IAF) and the frontal alpha asymmetry (FAA) measures computed from Muse data were consistent with those from a research-grade EEG system (Cannard et al., 2021); researchers successfully classified perceived mental stress level using the theta-band PSD from Muse (Arsalan et al., 2019). However, mindfulness-related EEG research using the Muse headset has generated mixed results. For example, one study using the Muse observed a significant increase in beta and gamma frequencies in the post-meditation sessions compared to pre-meditation (Karydis et al., 2018). Another study using the “calm score” computed by the Muse app (a proposed proxy for mindfulness), however, failed to observe “calm score” changes in participants after a 1-month meditation intervention. Additionally, some researchers have reported findings where the “calm score” did not reflect participants’ increased trait mindfulness (Acabchuk et al., 2020).

Portable, wireless EEG headsets are increasingly used to conduct social neuroscience research in naturalistic settings, and specifically in so-called hyperscanning studies—studies that simultaneously measure the brain activity of multiple people interacting with each other. Using a range of metrics to quantify inter-brain connectivity (Ayrolles et al., 2021), inter-brain coupling has been linked to a variety of factors during both verbal and non-verbal social tasks (Czeszumski et al., 2020). Inter-brain coupling has been associated with prosociality in various contexts. For example, inter-brain coupling studies using EEG have shown higher synchrony for couples than strangers in natural conversation and motor coordination tasks (Kinreich et al., 2017; Djalovski et al., 2021), in social coordination and cooperation (Mu et al., 2017; Barraza et al., 2020), and in teams with better collective performance (Reinero et al., 2021). During social interactions outside of laboratory environments, our group has previously found that inter-brain coupling is linked to social closeness, personal distress, and shared social attention (Dikker et al., 2017, 2021).

In sum, the first aim of the present study was to investigate whether laboratory findings on the neural correlates of mindful awareness replicate during naturalistic social interaction in an EEG device that has been explicitly associated with mindfulness meditation. Specifically, we asked if more mindful individuals exhibited enhanced EEG alpha (8–12 Hz) and theta (4–8 Hz) power during face-to-face social engagement (Takahashi et al., 2005). The second aim of this research was to capture possible “multi-brain” neural correlates of mindful awareness during naturalistic interaction. We investigated whether inter-brain coupling correlates with mindful awareness during naturalistic social settings, building on a growing body of research on mindfulness on the one hand, and social neuroscience research using portable EEG systems on the other.

## Materials and methods

### Participants

We collected data from participants who partook in the Mutual Wave Machine exhibition at Espacio Telefónica in Madrid, Spain (2019; see Study Setup). 554 individuals participated in the study, including 271 females, 245 males, and three individuals who identified as “other.” Participants’ ages ranged from 12 to 81 years, with an average of 33.8 years. After removing data with poor quality, we ended up with 379 participants for the PSD analysis, and 62 dyads (124 individuals) for inter-brain analysis (see Data Analysis). For inter-brain analysis, we retained 22% of participants, which is a lower retention rate than the previous iteration’s 39% (Dikker et al., 2021). This could be explained by the employment of the 4-channel EEG headset rather than 16 channels in the previous study (see Discussion).

Participants completed the questionnaires and consent forms in Spanish. They were informed that the primary purpose of participation was the art experience, but their data would be used for research in the future. Participation was voluntary and without monetary compensation. Individual written informed consent was obtained before the session.

### Study setup

This study was conducted as part of the participatory art installation Mutual Wave Machine where pairs of participants interacted naturally in a museum setting. This setup allowed us to study real-world face-to-face social interactions in a large population of participants recruited outside of the traditional research subject pool.

Museum visitors freely interacted with each other while their EEG was recorded using the Muse, a four-electrode wireless EEG system (Krigolson et al., 2017). In the current study, we recruited visitors at the Mutual Wave Machine exhibition at Espacio Telefónica in Madrid, Spain. Participants could participate either in pairs or individually to be paired with others. The artwork featured two shell-like structures enclosing the two participants facing each other, with visual projection on the shells and auditory feedback. EEG headsets were applied while participants completed a consent form and pre-experiment questionnaire. They were told the purpose of the work is to investigate whether being on the same “brain wavelength” related to their subjective feelings of “being in sync,” and that the brightness of the visual feedback reflected their synchrony level in real time. They were encouraged to try different strategies to achieve more synchrony. Note that there is evidence in past iterations that suggests being well-informed about the experiment increases pairs’ synchrony (Dikker et al., 2021). This increase, however, persisted even when the neurofeedback signal was a sham, suggesting the “placebo effect” of the knowledge about the experiment. We thus adopted this protocol to observe synchrony in the most encouraging setting.

The interaction typically lasted 10 min. Real-time inter-brain power correlations were calculated and used to generate visual feedback for the participants as part of the 10-min experience (Chen et al., 2021). Specifically, EEG data collected from the pair was processed in 6-s windows in real time. Both data streams were filtered into four frequency bands using FFTW (www.fftw.org; delta: 1–4 Hz; theta: 4–7 Hz; alpha: 7–12 Hz; beta: 12–30 Hz), and then Hilbert transformed to derive their instantaneous spectral power. Inter-brain synchrony was then calculated as the Pearson Correlation coefficient of the pairs’ instantaneous spectral power of particular frequency bands. Note that in some of the previous iterations, we took the effort to validate the EEG signal through eyes open/closed and up/down control experiments, which we did not perform in the current study due to practical limitations. Instead, we adopted strict data exclusion criteria semi-automatically (see EEG preprocessing).

Before and after the session, participants were asked to complete questionnaires for their affective traits and states (see Materials).

### Materials

All participants were asked to complete short questionnaires both before and after the session, addressing their relationship to each other, mood, and personality traits. Relationship measures include questions about relationship duration and social closeness. Affective personality trait measurement consisted of (a) a revised 14-item version of the Interpersonal Reactivity Index after the session (Davis and Others, 1980), including the subscales Personal Distress (e.g., “When I see someone who badly needs help in an emergency, I go to pieces”) and Empathic Concern (e.g., “I often have tender, concerned feelings for people less fortunate than me”). Internal reliability for each scale of the IRI was (<0.90), but it is in line with the literature: PD with a Cronbach’s alpha of 0.74 and EC with 0.74; and (b) the MAAS (Brown and Ryan, 2003), consisting of 15 items measuring one’s awareness of what is taking place at the present (e.g., “I could be experiencing some emotion and not be conscious of it until sometime later.”). Both questionnaires were answered on a five-point Likert scale ranging from “Does not describe me well” to “Describes me very well.” Social closeness was assessed using the Inclusion of the Other in the Self (IOS) Scale, a pictorial measure of closeness with two overlapping circles representing the self and the other (Aron et al., 1992). Participants also completed a shortened version of the Positive and Negative Affect Schedule (PANAS-X; (Watson and Clark, 1994), which was not analyzed here because the purpose of the study was to investigate the (inter-brain) neural correlates of mindful awareness.

This study focuses on mindful awareness, one facet of the self-report trait mindfulness (Baer et al., 2006). We used the Mindful Attention Awareness Scale (MAAS), a standardized questionnaire designed to assess the open awareness of the present moment (Brown and Ryan, 2003). The MAAS has been widely applied and shown to successfully probe specific aspects of mindfulness, such as acting with awareness (Coffey and Hartman, 2008), perceived inattention (Van Dam et al., 2010), and burnout and engagement (Kotzé and Nel, 2016). Due to the limitation of the setup, we used the short MAAS questionnaire (7 out of the 10 questions) instead of more comprehensive mindfulness measures such as the Five Facet Mindfulness Questionnaire (FFMQ; Baer et al., 2006) which contains 38 questions, longer than what we could fit in during the limited session each person had in the museum. Since MAAS only focuses on the awareness factor of the FFMQ (other factors are observing, describing, non-judging and nonreactivity), our finding addresses only mindful awareness, rather than mindfulness in general.

Before the session, participants completed Relationship measures, Empathic Concern part of the IRI, the MAAS scale, and the IOS scale. After the session, participants completed the Empathic Concern as well as the Personal Distress parts of the IRI, the IOS scale, and the PANAS-X questionnaire. Note that there was a risk of demand characteristics since MAAS was measured beforehand. However, the inter-brain synchrony was measured when participants were already encouraged to connect, so we believe demand characteristics due to MAAS testing is only secondary to such effects, if they exist.

### Data analysis

#### Personality metrics

After removing incomplete and incorrect data entries, 475 individuals’ answers were preserved. For the purpose of this study, the following metrics were analyzed: MAAS score, social closeness scale, Personal Distress, Empathic Concern, sex, age. To investigate which trait measure was related to mindful awareness, we constructed a multiple linear regression analysis using Personal Distress, Empathic Concern, age, and social closeness scale as predictors, and the MAAS score as the predicted variable.

#### EEG preprocessing

The initial dataset consisted of 277 pairs of ~10-min recordings. First, EEG data files were removed if files were not readable (4 pairs), the two EEG files were misaligned (119 pairs), or subjects’ self-reported information was missing (57 pairs). Each individual EEG dataset was then bandpass filtered from 0.1 to 30 Hz and segmented into 1-s epochs (“pseudo trials”). Bad channels were manually rejected upon visual inspection: since frontal channels (Fp1, Fp2) were noisier and more often removed than temporal channels (TP9, TP10), our data is mainly driven by the temporoparietal channels. We used the Python package “autoreject” (Jas et al., 2017) to remove epochs with movement artifacts and eye blinks, followed by manually checking the automatic selection and correction procedure. This resulted in the additional exclusion of 20 pairs due to poor data quality. Note that for inter-subject connectivity analyses, we only preserved temporally overlapping epochs that “survive” the preprocessing for both participants in each pair: unmatched epochs were removed. For individuals’ spectral power analyses, we used all the artifact-free epochs, regardless of the participants’ partners’ data, and removed participants with less than 60 clean epochs (60 s). Lastly, datasets with less than 50 remaining epochs (50 s) after these preprocessing steps were excluded from further analysis (15 pairs removed). These preprocessing steps resulted in 62 pairs (124 individuals) for the intersubject connectivity analysis, and 379 individuals for the spectral power analysis.

After preprocessing, we performed the short-time Fourier transform on the 1-s epochs, using a Hanning window with a one-sample step size, resulting in complex spectral coefficients of 1 Hz resolution from 1 to 30 Hz.

#### Individual PSD analysis

Individual PSD was computed from the preprocessed, epoched data (see previous section). We applied Welch’s method to estimate PSD per epoch from 1 to 30 Hz and averaged the result across all epochs and channels. The result was one PSD value per frequency for each participant. We then used cluster-based permutation analysis to investigate whether the PSD values significantly correlated with participants’ mindful awareness.

#### Inter-brain coupling analysis

Inter-brain coupling was calculated using Circular Correlation Coefficient (CCorr), which is a phase synchrony measure that is argued to be robust to spurious synchronization (Burgess, 2013; Goldstein et al., 2018). CCorr was computed between corresponding channels in the dyad, and then averaged across channel pairs.

To capture slower, transient information throughout the time series, the epoched complex coefficients were concatenated before the correlation (henceforth referred to as “concatenated”), resulting in two discontinuous complex series from the pair. CCorr was then computed by correlating the angular component of the two concatenated series for each pair with respect to all four channels using the Python package Astropy (Astropy Collaboration et al., 2018). The computation is demonstrated in eq. 1.1 (Circular Correlation and Regression, 2001), where *X* and *Y* are concatenated series from a certain frequency bin, and n represents the total number of time points (e.g., if 100 epochs are preserved, there are 256 Hz × 100 s = 25,600 time points). We used 1 Hz frequency bins ranging from 1 to 30 Hz. Following previous analyses of similar datasets, we also used a second metric, concatenated Projected Power Correlation (PPC; Hipp et al., 2012; Dikker et al., 2021) demonstrated in eq. 1.2–5, where $X\left(t,f\right)$ and $Y\left(t,f\right)$ are the concatenated complex coefficients at frequency f . First, the projection of $Y\left(t,f\right)$ on $X\left(t,f\right)$ is removed, leaving only the part of Y that’s orthogonal to X , i.e., $Y_{⊥X}\left(t,f\right)$ , and the same computation was done for $X\left(t,f\right)$ , resulting in $X_{⊥Y}\left(t,f\right)$ (eq. 1.2). Second, we computed the correlation between | $X\left(t,f\right)$ | and $Y_{⊥X}\left(t,f\right)$ , and | $Y\left(t,f\right)$ | and $X_{⊥Y}\left(t,f\right)$ respectively, and then averaged the two values as our PPC per frequency bin.

To investigate the difference between concatenating epochs versus averaging epochs, we also applied the same method to the epoched complex coefficients without concatenating (henceforth referred to as “epoched”). In such “epoched CCorr” or “epoched PPC,” the same calculation was done to individual epochs, and the result was an average of CCorr values across epochs. These exploratory results can be found in Supplementary Figures S1, S2.


(1.1) $$r_{\mathrm{circular}}=\frac{\sum_{i=1}^{n}\sin\left(x_{i}-\underline{x}\right)\sin\left(y_{i}-\underline{y}\right)}{\sqrt{\sum_{i=1}^{n}\sin{\left(x_{i}-\underline{x}\right)}^{2}\sum_{i=1}^{n}\sin{\left(y_{i}-\underline{y}\right)}^{2}}}$$

(1.2) $$Y_{⊥X}\left(t,f\right)=\mathrm{imag}\left(Y\left(t,f\right)\cdot \frac{X^{*}\left(t,f\right)}{\left|X\left(t,f\right)\right|}\right){\hat{e}}_{⊥X}$$

(1.3) $$X_{⊥Y}\left(t,f\right)=\mathrm{imag}\left(X\left(t,f\right)\cdot \frac{Y^{*}\left(t,f\right)}{\left|Y\left(t,f\right)\right|}\right){\hat{e}}_{⊥Y}$$

(1.4) $${\hat{e}}_{⊥X}\left(t,f\right)=\frac{iX\left(t,f\right)}{\left|X\left(t,f\right)\right|}$$

(1.5) $${\hat{e}}_{⊥Y}\left(t,f\right)=\frac{iY\left(t,f\right)}{\left|Y\left(t,f\right)\right|}$$

#### Cluster-based permutation analysis for correlation

The following procedure applies to both the individual PSD and pairs’ inter-brain coupling, in relation to either participants’ mindful awareness or pairs’ average mindful awareness, respectively. Interbrain synchrony is often computed using a variety of methods and thus is only meaningful in contrasting conditions with statistical analyses (Ayrolles et al., 2021). Therefore, we adopted the cluster-based permutation technique, a non-parametric and data-driven method to compare conditions.

To investigate the relationship between mindful awareness and inter-brain coupling, we computed the Pearson Correlation Coefficient between pairs’ MAAS score and connectivity metrics in every frequency bin, using a cluster-based nonparametric test to correct for multiple comparisons. The protocol is adapted from Dikker et al. (2021). First, Pearson correlation coefficients were computed between every frequency bin and pairs’ average MAAS scales, generating 30 correlation values. Correlation significance thresholds r_upper and r_lower were then determined by choosing the 97.5th and 2.5th percentile of the 30 correlation values, respectively. Then the random permutation procedure started with randomly shuffling the behavioral variable (e.g., MAAS scale), and computing correlation values for each frequency bin. Correlation values higher than r_upper or lower than r_lower were marked as significant in this permutation, and significant correlation values that were adjacent in frequencies were identified as clusters. For each cluster, we extracted the cluster size as our cluster statistics. When there was more than one cluster, the maximum cluster size was chosen. This random permutation procedure was repeated 2000 times, generating a distribution of cluster statistics, of which the 95th percentile was determined as the significant cluster threshold. Last, from the actual correlation values, we identified clusters using a value of p threshold of 0.1 and compared their sizes to the significant cluster threshold. The Monte-Carlo value of p was then calculated from the percentile score of the actual cluster size in the cluster statistic distribution. A Monte-Carlo value of p lower than the significant threshold 0.05 would mean the actual cluster size is larger than 95% of the random distribution of cluster sizes.

We applied the same procedure to correlate mindful awareness with individual PSD values, replacing the pairs’ average MAAS scale with individuals’ MAAS scale.

## Results

### Individuals’ EEG spectral power does not predict mindful awareness

**Figure 1:**
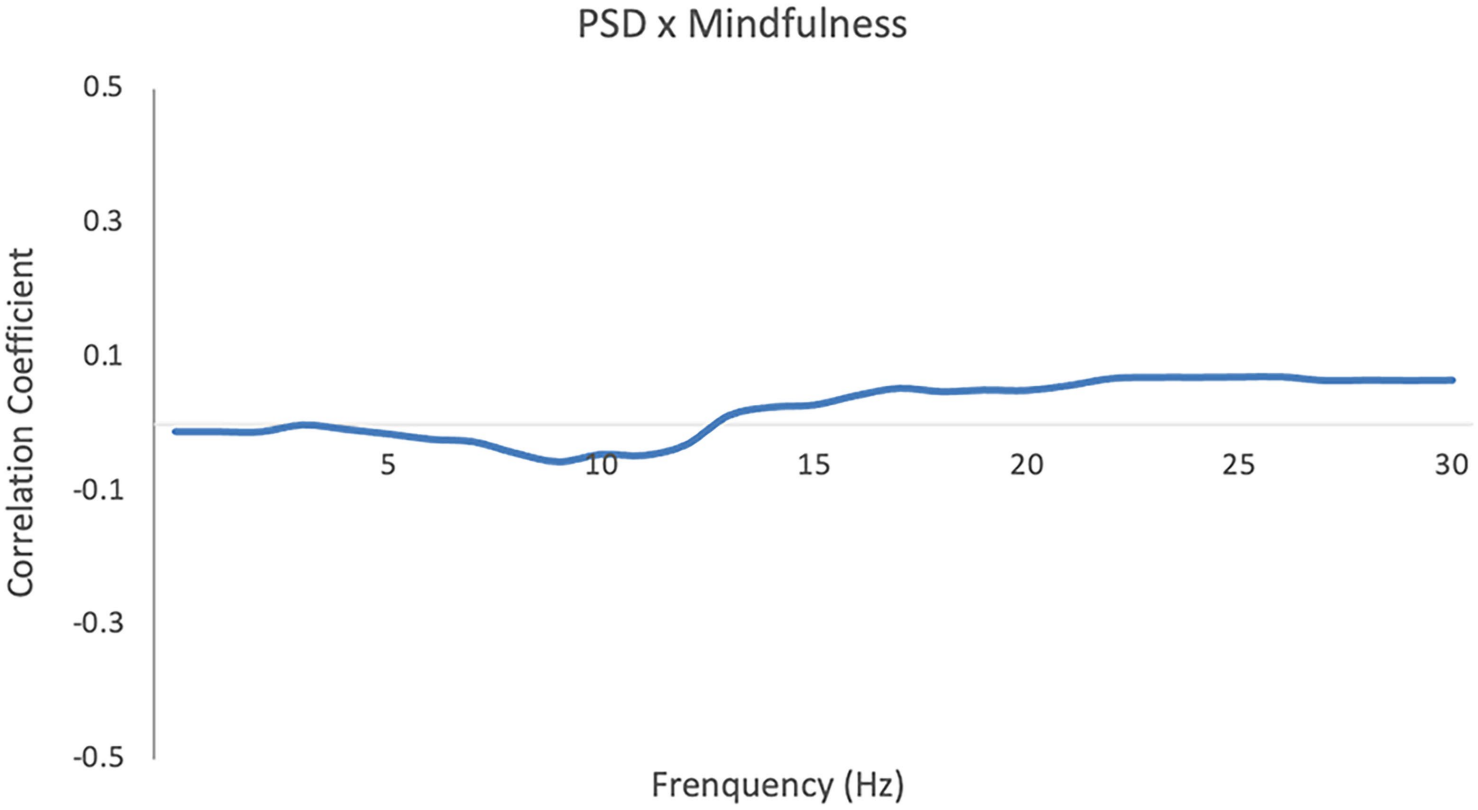
Pearson correlation coefficient between individuals’ MAAS score and their power spectral density (PSD). Cluster-based permutation analysis of the correlation coefficients showed no significant clusters (*p* = 0.92).

Contrary to prior findings illuminating neurobiological changes related to mindfulness in laboratory contexts, i.e., a global increase in alpha and theta power during various kinds of meditations (Takahashi et al., 2005; Lomas et al., 2015; Lee et al., 2018), we found no significant correlation between individuals’ power spectral density during social interaction and their MAAS scales (Figure 1).

### Inter-brain coupling is correlated with dyads’ mindful awareness

**Figure 2:**
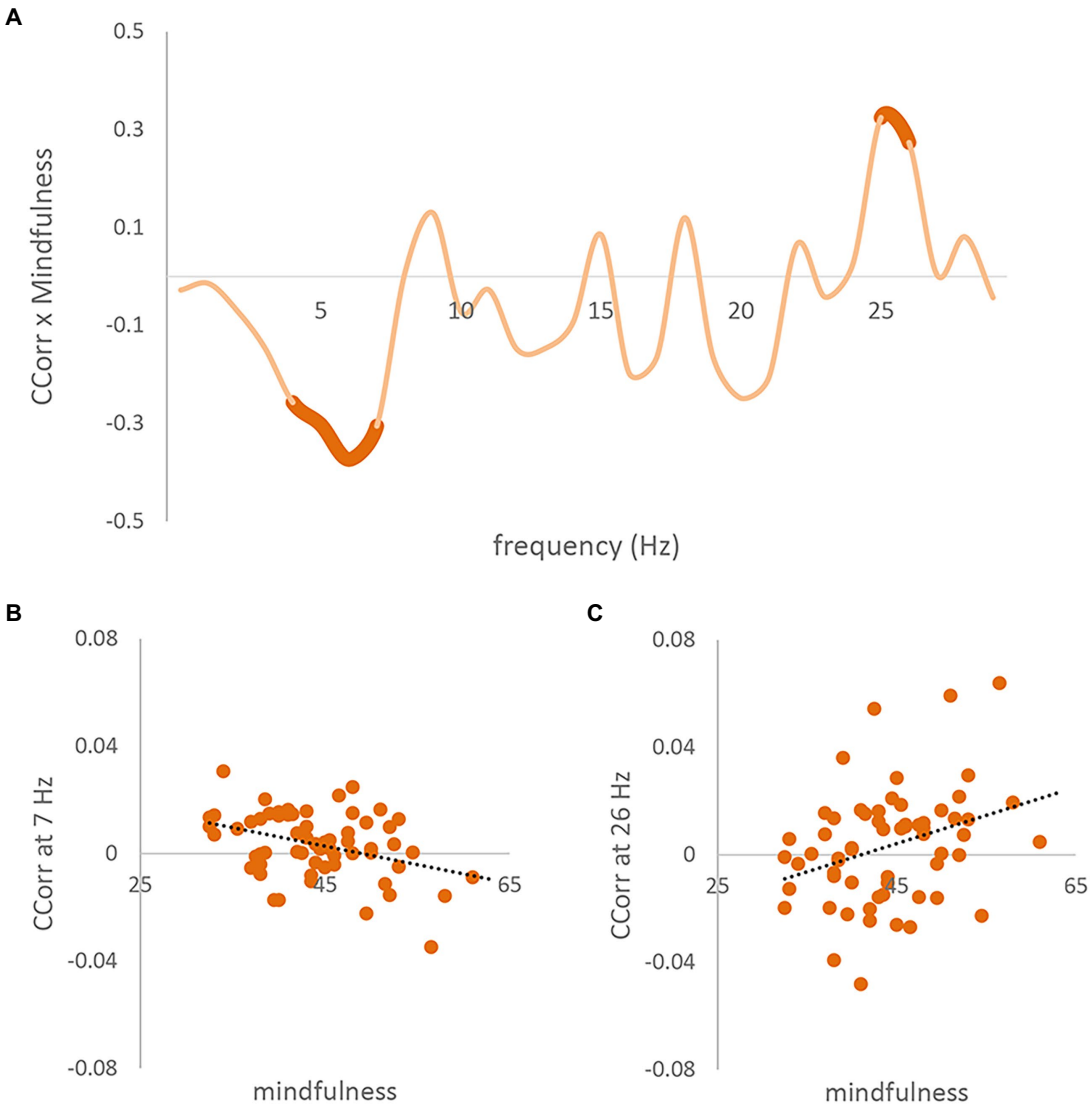
Correlation results between trait mindful awareness and CCorr. **(A)** Pearson correlation coefficients (*y*-axis) between pair-averaged MAAS and inter-brain coupling (CCorr), for each 1-Hz frequency bin from 1 to 30 Hz (*x*-axis). Two significant clusters (monte-carlo *p* = 0.002) are highlighted in bold. **(B)** Scatter plot between CCorr at 7 Hz and pair-averaged MAAS. The dotted line is the linear regression line. [r(62) = −0.373]. **(C)** Scatter plot between concatenated CCorr at 26 Hz and pair-averaged MAAS scale [r(62) = 0.325].

The cluster-based permutation analysis showed that pairs’ mindful awareness predicted inter-brain coupling (CCorr; Monte-Carlo value of *p* < 0.001). Specifically, as can be seen in Figure 2B, there were two clusters where the CCorr theta band (5–8 Hz) cluster shows a negative correlation between CCorr and subjects’ MAAS scales [Figure 2B, circular correlation coefficient at 7 Hz; r(62) = −0.373], and the high beta band (26–27 Hz) cluster shows a positive correlation [Figure 2C, circular correlation coefficient at 26 Hz; r(62) = 0.325]. Figure 2A shows the Pearson Correlation Coefficient between the MAAS and CCorr at every frequency from 1 to 30 Hz, with significant clusters marked with bold lines.

### Personal distress predicts mindful awareness

**Figure 3:**
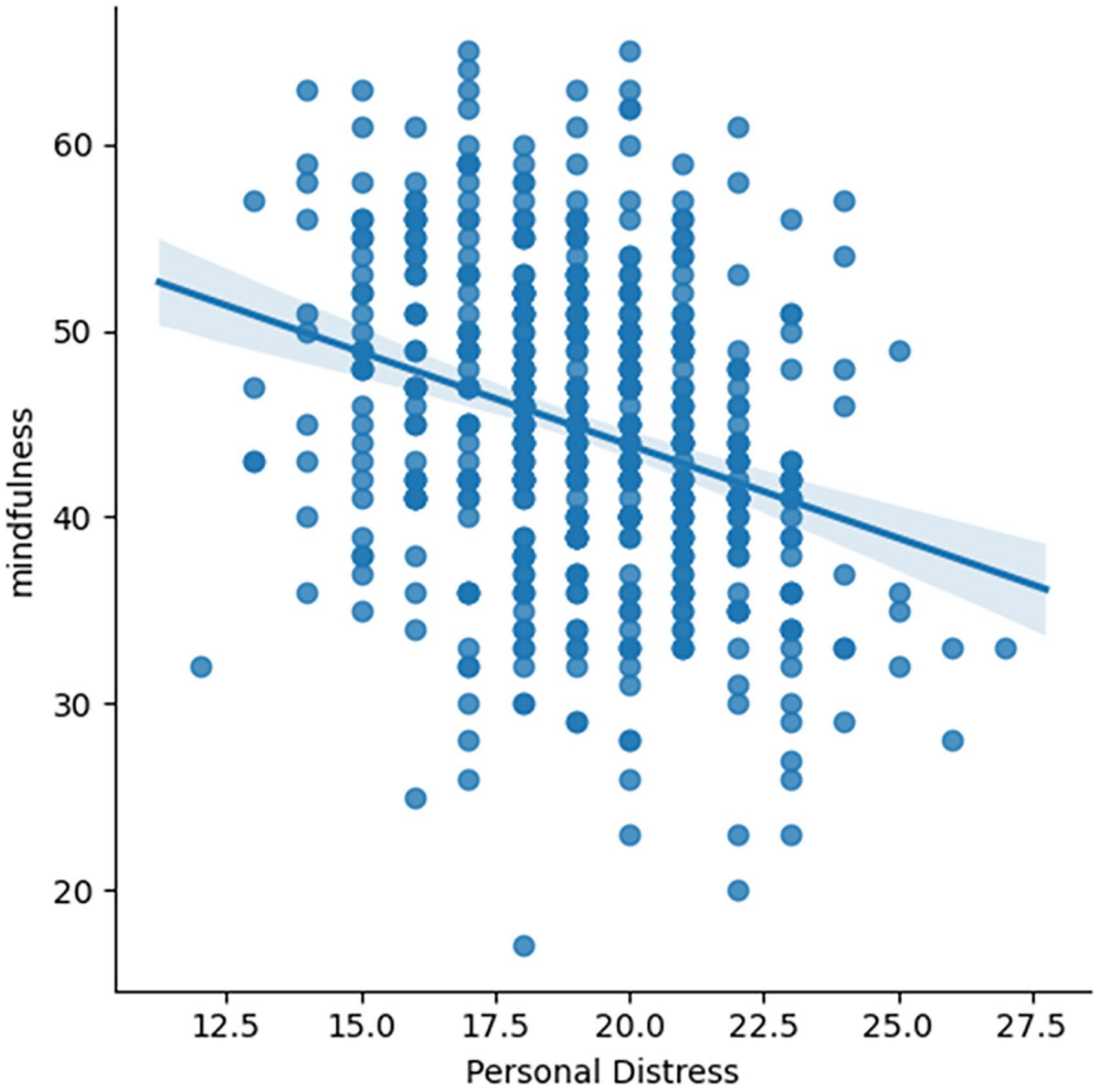
Correlation between personal distress and MAAS. Individuals’ personal distress is negatively correlated with MAAS scale [t(475) = −5.493, *p* < 0.001].

The regression analysis showed Personal Distress as the only significant predictor among sex, age, Empathic Concern and Social Closeness [t(475) = −5.493, *p* < 0.001; Figure 3] for trait mindful awareness (for the full results, please see Supplementary Table S1). Individuals with lower personal distress reported higher MAAS scale.

## Discussion

This study investigates the neural correlates of mindful awareness in naturalistic face-to-face social interactions. Our study investigated the relationship between mindful awareness and prosociality on both the psychometric and neurobiological level. The questionnaire results showed that trait mindful awareness was associated with lower personal distress. The EEG results showed that neural correlates of mindful awareness during social interaction were found in interbrain synchrony, but not in individual EEG power changes.

### Intra- vs. inter-individual neural correlates of mindful awareness

Contrary to previous studies, we did not find a relationship between individual brain activity (power spectral density) and mindful awareness. There are a few possible explanations for this null effect. First, past studies investigating EEG power and mindfulness have been focusing on different types of mindfulness correlates from ours: power changes in individuals have been observed only in meditative states (Lutz et al., 2004; Takahashi et al., 2005), and functional changes in other controlled lab tasks are observed with fMRI (Brefczynski-Lewis et al., 2007; Farb et al., 2007; Grant et al., 2011; Taylor et al., 2011; Allen et al., 2012; Hasenkamp and Barsalou, 2012; Lutz et al., 2013; Mascaro et al., 2013) or in event-related potentials with EEG (Brown et al., 2013; Wong et al., 2018). Second, using a four-channel portable EEG system in a noisy, less controlled setting might also have contributed to a null result. It is important to reiterate, however, that we *did* find inter-brain correlates of mindful awareness during social interaction.

This is not the first study to report a discrepancy between intra- and inter-brain neural correlates: other hyperscanning studies have similarly found that a multi-brain approach captures neural correlates of social behaviors that are not observed in individuals (Simony et al., 2016; Balconi et al., 2017; Davidesco et al., 2019; Dikker et al., 2021). For example, in one study an inter-brain network but not individual brain activity predicted players’ strategy in prisoner’s dilemma (Fallani et al., 2010), and inter-brain coupling but not individual alpha power nor intra-brain synchrony predicted students’ performance during lessons (Davidesco et al., 2019). Under the rationale that online mutual interaction is “a complex nonlinear system that cannot be reduced to the summation of effects in single isolated brains” (Koike et al., 2015), our study further validates a multi-brain approach in complex social tasks by extending it to a naturalistic setting and portable EEG systems.

Note that in previous iterations of the current study’s experimental setup, the Mutual Wave Machine, we found inter-brain coupling was correlated with pairs’ relationship duration and with their affective personalities (social closeness and perspective taking) using the EMOTIV portable EEG system. The current study intends to validate the easier-to-use EEG system Muse, focusing on a different affective trait, mindful awareness. Although we did not replicate previous findings about social closeness and perspective taking, it could be due to the significant difference in experimental setup and data analysis methods between the current study and past iterations using EMOTIV.

### Mindful awareness and empathy measures

Our questionnaire results showed a negative correlation between trait mindful awareness and personal distress, but not between trait mindful awareness and empathic concern. In line with previous research, this result suggests that mindful awareness might alleviate the negative consequences of empathy, but not necessarily contribute to empathic feelings: Previous research has suggested a complicated relationship between mindfulness and empathy, especially when empathy is dissected as a multidimensional construct (Davis and Others, 1980). Measuring overall self-reported empathy, some studies have reported a positive correlation between trait mindfulness and empathy (Beitel et al., 2005; Dekeyser et al., 2008; Greason and Cashwell, 2009), and mindfulness-based stress reduction (MBSR) training has been shown to increase participants’ self-reported empathy (Shapiro et al., 1998). However, other studies did not find effects of mindfulness-based training on self-reported empathy, empathic concern, or emotion recognition (Galantino et al., 2005; Lim et al., 2015). Specifically, in an eight-week MBSR training for nursing students, self-reported personal distress decreased, whereas self-reported empathic concern and perspective taking did not change (Beddoe and Murphy, 2004).

Distinct from empathic concern, personal distress is a self-oriented negative feeling that is often associated with reduced perspective taking and compassion fatigue when witnessing others’ suffering, and can be unrelated to prosocial behaviors (Pulos et al., 2004; FeldmanHall et al., 2015). Thus, our finding that trait mindful awareness is negatively correlated with personal distress but not related to empathic concern, is in line with previous work. Such empirical evidence supports the theory that mindful awareness is a vital part of self-compassion, which contributes to the resiliency against emotional fatigue (Figley, 1995; Neff, 2003; Thomas, 2012).

### Relationship between trait mindful awareness and inter-brain coupling

We observed a positive correlation between inter-brain coupling and mindful awareness in the beta frequency range, but a *negative* correlation in the theta frequency range. This finding seems puzzling in light of past studies reporting a positive relationship between inter-brain coupling and prosocial behavior and prosocial traits (Valencia and Froese, 2020). However, recent work has pushed back on the leading assumption that more synchrony is always ‘better’ (for review, see Mayo and Gordon, 2020). For example, multiple studies have found cognitive downsides of behavioral synchrony, such as insecure attachment (Feniger-Schaal et al., 2016), worse performance in cooperative problem solving (Abney et al., 2015; Wallot et al., 2016), and decreased self-regulation (Galbusera et al., 2019). Physiological research has also yielded mixed results about the link between synchrony and couples’ relationships as well as parent-infant engagement (Timmons et al., 2015; Wass et al., 2019). In their review, Mayo and Gordon (Mayo and Gordon, 2020) proposed situating synchrony in an interpersonal system that contains both collective and independent behaviors and taking into account both the synchronization and segregation aspects inherent to synchrony. While neural studies overwhelmingly report positive relationships between inter-brain synchrony and social factors, there too, some studies pointed to the complexities of such relationships. For example, Goldstein et al. (2018) found that, during hand holding, romantic partners’ inter-brain coupling (CCorr) was *negatively* correlated with analgesia of the target person upon pain stimulation, and *positively* correlated with empathic accuracy of the partner. Separate examination of the effect of pain and touch, however, suggested distinct brain-coupling components associated with the experience of pain and the empathy for pain. The relationship between inter-brain synchrony and mindful awareness, a personality trait that entails a mixture of cognitive properties, may also correspond to multiple processes and require further investigation.

## Conclusion

This study used consumer-grade portable EEG (Muse) and asked how trait mindful awareness and prosociality manifest itself in neural responses during naturalistic dyadic face-to-face social interactions. Neural correlates of mindful awareness during social interaction were evident in inter-brain coupling, but we did not replicate previous studies showing individual EEG power changes as a function of mindful awareness using a consumer-grade EEG system. In addition, the directionality and signature of the relationship between mindful awareness and inter-brain coupling varied by frequency and by how inter-brain coupling was computed. Together, our findings are suggestive of a complex relationship between mindful awareness and inter-brain coupling, while at the same time raising a cautionary note about methodological approaches in hyperscanning research.

## Data availability statement

The raw data supporting the conclusions of this article will be made available by the authors upon request, without undue reservation.

## Ethics statement

Ethical review and approval was not required for the study on human participants in accordance with the local legislation and institutional requirements. Written informed consent to participate in this study was provided by the participants themselves, or their legal guardian/next of kin for underage participants.

## Author contributions

PC, SD, and UK: study conception and design. SD: data collection. PC: analysis and interpretation of results and draft manuscript preparation. All authors contributed to the article and approved the submitted version.

## Funding

This project was funded by a grant from Lundbeckfonden #R291-2018-1462 (UK), The Netherlands Organization for Scientific Research grant #406.18.GO.024, and The Dutch Creative Industries Fund, and Fundación Telefónica (SD).

## Conflict of interest

The authors declare that the research was conducted in the absence of any commercial or financial relationships that could be construed as a potential conflict of interest.

## Publisher’s note

All claims expressed in this article are solely those of the authors and do not necessarily represent those of their affiliated organizations, or those of the publisher, the editors and the reviewers. Any product that may be evaluated in this article, or claim that may be made by its manufacturer, is not guaranteed or endorsed by the publisher.

## Supplementary material

The Supplementary material for this article can be found online at: https://www.frontiersin.org/articles/10.3389/fpsyg.2022.915345/full#supplementary-material
