# Supplementary material for: Trait mindful awareness predicts inter-brain coupling but not individual brain responses during naturalistic face-to-face interactions
Source: Front Psychol. 2022 Sep 30;13:915345. doi: 10.3389/fpsyg.2022.915345 (PMC9561904; doi:10.3389/fpsyg.2022.915345)
Supplement: Supplementary file 1 [file Data_Sheet_1.PDF]

Supplementary Material

1 supplementary data

Source	B	SE B	t	p
sex	0.264	0.732	-0.323	0.718
(post) Personal Distress	-0.867	0.155	-5.608	<0.001
(post) Empathic Concern	0.191	0.137	1.390	0.165
(avg) Social Closeness	0.146	0.239	0.611	0.541
age	0.057	0.031	1.869	0.062

Supplementary Table S1. Regression table for predicting individuals' mindfulness scale. Both Personal Distress Scale and the Empathic Concern Scale were calculated from post-session answers to the Interpersonal Reactivity Index (IRI; Davis & Others, 1980); social closeness was an average of pre- and post-session answers to the Inclusion of the Other in the Self (IOS) Scale (Aron et al., 1992).

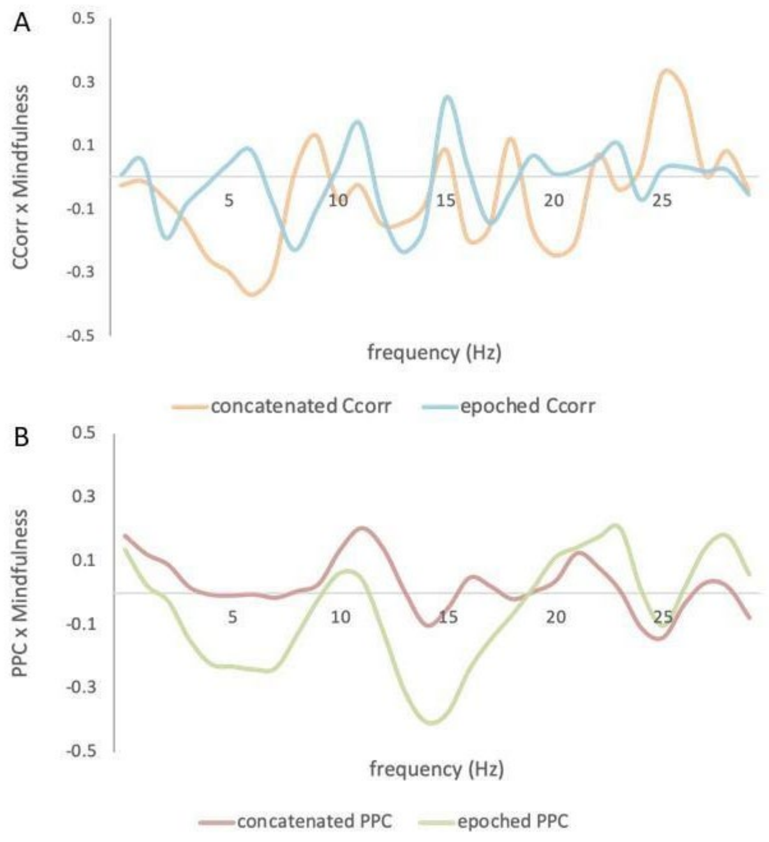

Supplementary Figure S1. Pearson correlation coefficient between pair-averaged mindfulness and inter-brain synchrony, computed with (**A**) epoched Circular Correlation (CCorr; Burgess, 2013) and concatenated CCorr and (**B**) epoched CCorr and concatenated Projected Power Correlations (PPC; Hipp et al., 2012). While the averaging method does not affect PPC trend significantly, it does in the case of CCorr, showing different and even opposite relationships at certain frequencies.

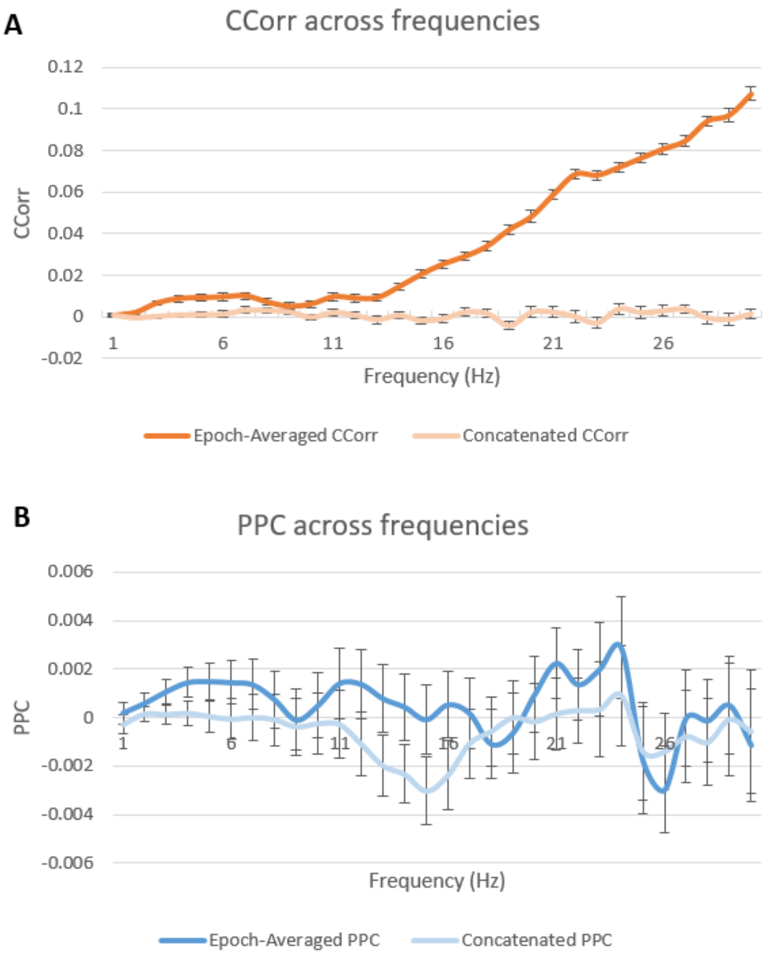

Supplementary Figure S2. Connectivity values averaged across subject pairs for each frequency bin. (**A**) epoched and concatenated Circular Correlation (CCorr; Burgess, 2013). (**B**) epoched and concatenated CCorr. As frequency increases, epoched CCorr values diverged significantly from concatenated CCorr, while epoched Projected Power Correlation (PPC; Hipp et al., 2012) and concatenated PPC stay relatively similar. This would explain the different correlation results in Figure S1, where epoched PPC and concatenated PPC had similar trends of correlation with mindfulness, whereas epoched CCorr showed different and sometimes opposite trends from concatenated CCorr. This interaction might be because phase synchrony captures much faster temporal information in epoched data compared to power synchrony as discussed in methods, and thus is more susceptible to window lengths. We thus suggest that phase synchrony computation should adopt frequency-dependent windowing or other methods to compensate for an increasing number of cycles as frequency rises in a fixed time period.

2 References

Aron, A., Aron, E. N., & Smollan, D. (1992). Inclusion of Other in the Self Scale and the structure of interpersonal closeness. *Journal of Personality and Social Psychology*, *63*(4), 596–612. https://doi.org/10.1037/0022-3514.63.4.596

Burgess, A. P. (2013). On the interpretation of synchronization in EEG hyperscanning studies: a

cautionary note. *Frontiers in Human Neuroscience*, *7*, 881. https://doi.org/10.3389/fnhum.2013.00881

Davis, M. H. & Others. (1980). *A multidimensional approach to individual differences in empathy*. https://www.uv.es/friasnav/Davis_1980.pdf

Hipp, J. F., Hawellek, D. J., Corbetta, M., Siegel, M. & Engel, A. K. (2012). Large-scale cortical correlation structure of spontaneous oscillatory activity. *Nature Neuroscience*, *15*(6), 884–890. https://doi.org/10.1038/nn.3101
